# The Role of Immune Checkpoint Blockade in the Hepatocellular Carcinoma: A Review of Clinical Trials

**DOI:** 10.3389/fonc.2021.801379

**Published:** 2021-12-08

**Authors:** Muhammet Ozer, Andrew George, Suleyman Yasin Goksu, Thomas J. George, Ilyas Sahin

**Affiliations:** ^1^ Department of Internal Medicine, Capital Health Medical Center, Trenton, NJ, United States; ^2^ Department of Chemistry, Brown University, Providence, RI, United States; ^3^ Department of Molecular Biology, Cell Biology & Biochemistry, Division of Biology and Medicine, Brown University, Providence, RI, United States; ^4^ Division of Hematology/Oncology, Department of Medicine, University of Texas Southwestern Medical Center, Dallas, TX, United States; ^5^ Division of Hematology/Oncology, Department of Medicine, University of Florida, Gainesville, FL, United States; ^6^ Division of Hematology/Oncology, Department of Medicine, University of Florida Health Cancer Center, Gainesville, FL, United States

**Keywords:** immune checkpoint blockade, immunotherapy, hepatocellular carcinoma (HCC), liver cancer (LC), clinical trials

## Abstract

The prevalence of primary liver cancer is rapidly rising all around the world. Hepatocellular carcinoma (HCC) is the most common type of primary liver cancer. Unfortunately, the traditional treatment methods to cure HCC showed poor efficacy in patients who are not candidates for liver transplantation. Until recently, tyrosine kinase inhibitors (TKIs) were the front-line treatment for unresectable liver cancer. However, rapidly emerging new data has drastically changed the landscape of HCC treatment. The combination treatment of atezolizumab plus bevacizumab (immunotherapy plus anti-VEGF) was shown to provide superior outcomes and has become the new standard first-line treatment for unresectable or metastatic HCC. Currently, ongoing clinical trials with immune checkpoint blockade (ICB) have focused on assessing the benefit of antibodies against programmed cell death 1 (PD-1), programmed cell death-ligand 1 (PD-L1), and cytotoxic T-lymphocyte- associated antigen 4 (CTLA-4) as monotherapies or combination therapies in patients with HCC. In this review, we briefly discuss the mechanisms underlying various novel immune checkpoint blockade therapies and combination modalities along with recent/ongoing clinical trials which may generate innovative new treatment approaches with potential new FDA approvals for HCC treatment in the near future.

## Introduction

Hepatocellular carcinoma (HCC) is a primary malignant disease of the liver. Primary liver cancer is the seventh most common malignancy and the second most frequent cancer-related death worldwide (1). The majority of HCC patients are diagnosed with unresectable or advanced disease and have a poor prognosis (2). Thus, clinicians and scientists are eager to find novel therapeutic approaches for HCC that effectively balance clinical benefit with toxicity. The main objective for early stage HCC is curing the disease, and traditional therapeutic methods mainly include surgery, liver translaplantation or other localized liver-directed therapies (e.g., trans-arterial chemoembolization [TACE] and radiofrequency ablation [RFA]). However, treatment options for the advanced stage HCC are relatively limited and palliative in nature (3).

Until recently, tyrosine-kinase inhibitors (TKIs) like oral sorafenib were the most common gold-standard treatment for advanced HCC. However, the IMbrave150 trial (NCT03434379) demonstrated that atezolizumab combined with bevacizumab resulted in better overall survival (OS) and progression-free survival (PFS), effectively replacing sorafenib as a new standard first-line treatment for advanced HCC, particularly for those with near normal liver function (4). In situations where atezolizumab with bevacizumab is contraindicated or not available, TKIs (preferably sorafenib or lenvatinib) may also be considered for first-line treatment of advanced HCC. Otherwise, alternative treatments including nivolumab and other agents with less data supporting their use may be considered, consistent with national guidelines (3). As second-line treatments, regorafenib and cabozantinib were shown to prolong survival compared with placebo and received approval from the United States Food and Drug Administration (FDA) for advanced HCC patients who previously received sorafenib (5, 6). Ramucirumab also showed efficacy after sorafenib among advanced HCC patients with AFP levels >400 ng/ml (7). Although the prognosis of HCC remains dismal, the recent developments in systemic therapies - particularly in immunotherapy and targeted therapies - have increased overall survival and the quality of life of patients.

In the near past, oncolytic immunotherapy has arisen as a promising approach for inhibiting tumor progression and metastasis (8). The justification of this approach relates to enhancing cellular or humoral immunity *via* activating tumor-specific immune responses and disrupting immune tolerance. Developments in this field have led to many FDA approvals of immune checkpoint inhibitors (ICI) as primary treatment options for several different solid and hematologic malignancies (9). Moreover, novel treatment combinations along with newly identified druggable targets are expected to expand the role of immunotherapy in the treatment of a variety of cancers in the coming years. Recent developments also suggest promising antitumor effects of immunotherapy in HCC, highlighting the importance of this treatment modality amongst an otherwise limited set of treatment options.

Immune checkpoint molecules mainly function to maintain immunotherapeutic balance and protect against uncontrolled immunity by preventing excessive activation of T cells (10). However, negative immune regulators may be overexpressed in tumors to escape immune surveillance. Therapeutic ICIs counteract this escape and reactivate tumor-specific T cells *via* suppressing checkpoint-mediated signals (11). Cytotoxic T-lymphocyte-associated antigen 4 (CTLA-4), programmed cell death protein 1 (PD-1), programmed cell death protein-ligand 1 (PD-L1), B and T lymphocyte attenuator (BTLA), V-domain immunoglobulin suppressor of T cell activation (VISTA), T-cell immunoglobulin and mucin domain 3 (TIM-3), lymphocyte activation gene-3 (LAG-3), and tumor necrosis factor receptor superfamily member 4 (OX40) are the main ICIs under investigation (12). Although it is a promising treatment modality for a variety of cancers, tumors often exhibit primary, adaptive, or acquired resistance to immunotherapy which might be intrinsic to the tumor cells or that may be influenced by their microenvironment (13). In addition to cytotoxic T cells, there are other essential mediators of immune homeostasis such as T regulatory cells (Tregs) (14), myeloid-derived suppressor cells (MDSCs) (15), regulatory dendritic cells (16), and NK cells (17) which also play an essential role in response to immunotherapy treatments.

The conditional FDA approval in 2017 of the anti-PD-1 antibody nivolumab to treat advanced HCC in patients who have been previously treated with sorafenib opened a new era of drug development for advanced HCC. Single-agent ICIs were found to provide clinical benefits in 15–20% of responders, however, biomarkers have failed to help identify this subgroup (18). Fortunately, there are multiple early and advanced stage clinical trials investigating the efficacy of combination therapies, including combining ICIs with TKIs or combining PD1/PDL1 axis inhibitors with CTLA4 inhibitors, which might change the landscape of HCC management for different stages in the near future. Current research progress in the treatment of HCC mainly include ICIs, tumor vaccines, and adoptive cell therapy. This paper reviews ongoing clinical trials of ICIs in HCC patients.

## Background of Immune Checkpoint Inhibitors (ICIs) in HCC

Tasuku Honjo identified PD-1 as an immune checkpoint molecule at Kyoto University in 1992 (19). Many years later, nivolumab targeting the PD-1 was approved for the treatment of patients with melanoma in 2014 as the first anti-PD-1 antibody. Consequently, the FDA granted approval to nivolumab for the treatment of non-small-cell lung cancer and kidney cancer in the USA. James Allison was the first to show that CTLA-4 is also a therapeutic target for cancer treatment (20). Ipilimumab, an antibody targeting CTLA-4, was subsequently developed and approved by the FDA as an anti-melanoma agent in the USA in 2011 (21). Together, these two pioneers were each awarded the Nobel Prize in Physiology or Medicine in 2018, reflecting the impact of their seminal work on the field of oncology (22).

The liver has a highly complex immune tolerance system driven by antigen-presenting cells (APCs), namely dendritic cells (DCs) and liver-specific APCs. These complex antigen presentation mechanisms require multiple costimulatory signals to achieve T-cell activation and clonal expansion. Meanwhile, APCs also produce additional signals for immune checkpoint molecules, limiting T cell hyperactivation. These suppressive signals from immune checkpoint molecules have an active role in maintaining tolerance and preventing unwanted immune responses. Malignant tumor environments disrupt normal suppressive signals and cause T-cell exhaustion, characterized by high expression of immune checkpoint molecules, impaired cytotoxicity, and low levels of effector cytokines, leading to chronic hyporesponsive immunity (23). Since immune checkpoint molecules seem responsible for immunotolerance, many clinical trials have been designed to confirm their function in HCC treatment.

PD-1 is mainly expressed in T cells, B cells, natural killer (NK) cells, mononuclear cells, and dendritic cells (24). PD-1 inhibitors activate immune cells by blocking the receptor binding of PD-L1 and PD-L2 (25). Well-known PD-1 inhibitors nivolumab and pembrolizumab have largely been investigated for immunotherapy in cancer patients. A non-comparative phase I/II CheckMate 040 trial of Nivolumab (NCT01658878) with 262 patients with advanced HCC showed the safety and efficacy of PD-1 inhibitor in treating HCCs (26). The objective response rate (ORR) was 20%, with a median OS of 15 months. Nivolumab activity was observed regardless of tumor PD-L1 expression, prior sorafenib exposure, and underlying etiology. Based on this study, in 2017, nivolumab received conditional approval as a second-line treatment of patients with advanced and metastatic HCC. To evaluate nivolumab further as a monotherapy, an open-label, multicenter, randomized Phase III Checkmate 459 trial comparing nivolumab with sorafenib (NCT02576509) as first-line therapy was conducted in patients with advanced HCC (27). At ESMO World Congress on Gastrointestinal Cancer 2020 Virtual, the study team presented long-term follow-up results. Although 1.5 months longer OS was observed with nivolumab, it did not meet the predefined threshold for significance with a minimum follow-up of 33.6 months. However, nivolumab had a more favorable and manageable safety profile with better preservation of liver function over time compared with sorafenib. Another anti-PD-1 antibody, pembrolizumab, has been studied in a multicenter, randomized Phase II KEYNOTE-224 study (NCT02702414) in sorafenib-refractory advanced HCC patients (28). The ORR was 17%, with a 1% complete response (CR), 16% partial responses (PR). Stable disease (SD) was achieved in 44% of patients, the median OS was 12.9 months, and the PFS was 4.9 months (28). With those results, the FDA approved pembrolizumab as a second-line agent in patients with HCC whose disease progressed under sorafenib treatment. The subsequent randomized, double-blind Phase III KEYNOTE-240 (NCT02702401) assessed pembrolizumab *versus* placebo in the second-line setting. Unfortunately, the HR for OS (0.781; 95% CI, 0.611 to 0.998; P 5.0238) and PFS (HR, 0.72; 95% CI, 0.57 to 0.90) did not reach prespecified statistical plan (29).

Anti-PD-L1 antibodies, durvalumab and avelumab, are currently undergoing active assessment in clinical trials. Durvalumab showed 10.3% ORR in Phase I/II study of advanced HCC patients following prior treatment with sorafenib (NCT01693562) (30). In a single-arm phase II study, avelumab was evaluated in patients with advanced HCC also previously treated with sorafenib (NCT03389126). After a median follow-up of 13.7 months, avelumab showed moderate efficacy (no CR, 10.0% PR, and 63.3% SD) and was well tolerated (no grade 4 adverse events and 23% grade 3 adverse events) (31). Other PD-1/PD-L1 trials, including tislelizumab (NCT03412773, NCT03419897) are currently in development as well.

CTLA-4 is mainly expressed by Tregs, activated T cells, and NK cells (32). CTLA-4 inhibitors accelerate T cell activation by preventing the binding of CTLA-4 to B7-1 and B7-2. One of the well-known actions of the anti-CTLA-4 antibody is to downregulate Tregs in the tumor microenvironment. Given that CTLA-4 is most strongly expressed on Tregs, the mechanism of action of anti- CTLA-4 antibodies may involve inhibition of Tregs activity. As the earliest immune checkpoint inhibitor therapy, a phase II study of the anti-CTLA-4 antibody tremelimumab (NCT01008358) showed a promising effect with a 17.6% partial response rate, and a 76% disease control rate in patients with advanced HCC whose disease progressed under sorafenib treatment (33). Current ongoing clinical studies are actively investigating the efficacy and safety of the anti-CTLA-4 antibodies ipilimumab and tremelimumab. Many ongoing clinical trials of monotherapy for ICIs in HCC are listed in **Table 1**.

**Table 1:** Ongoing clinical trials of monotherapy for immune checkpoint inhibitors in hepatocellular carcinoma (HCC).

Agent	Phase	Clinicaltrial.gov	Trial Name	Patient characteristics	Primary Outcome
Nivolumab	Phase 2	NCT03630640	NIVOLEP	Neoadjuvant and adjuvant treatment in patients treated by Electroporation	Local RFS
Nivolumab	Phase 3	NCT03383458	CheckMate 9DX	Patients who received curative resection or ablation	RFS
Nivolumab	Phase 1 & 2	NCT01658878	CheckMate-040	Systemic treatment naïve patients with advanced HCC	ORR, AEs
Pembrolizumab	Phase 1	NCT02595866	–	Patients with HIV and relapsed, refractory or disseminated HCC	AEs
Pembrolizumab	Phase 2	NCT02702414	KEYNOTE-224	Patients with advanced HCC	ORR
Pembrolizumab	Phase 2	NCT03163992	–	2^nd^ line treatment after Sorafenib failure	ORR
Pembrolizumab	Phase 3	NCT03867084	KEYNOTE-937	Patients with HCC who has complete radiologic response after resection or local ablation	RFS, OS
Pembrolizumab	Phase 1 & 2	NCT02940496	–	2^nd^ line treatment in advanced HCC	DLTs
Pembrolizumab	Phase 2	NCT03419481	–	Hep B related HCC	RR
Pembrolizumab	Phase 3	NCT03062358	KEYNOTE-394	2^nd^ line treatment in Asian patients	OS
Toripalimab	Phase 2 & 3	NCT03859128	JUPITER-04	Adjuvant therapy following radical resection	RFS
Tislelizumab	Phase 3	NCT03412773	RATIONALE-301	1^st^ line treatment in patients with advanced HCC	OS
Tislelizumab	Phase 2	NCT03419897	RATIONALE-208	Patients who have been previously systemically treated	ORR

## Combination Therapy With Immune Checkpoint Inhibitors

**Figure 1:**
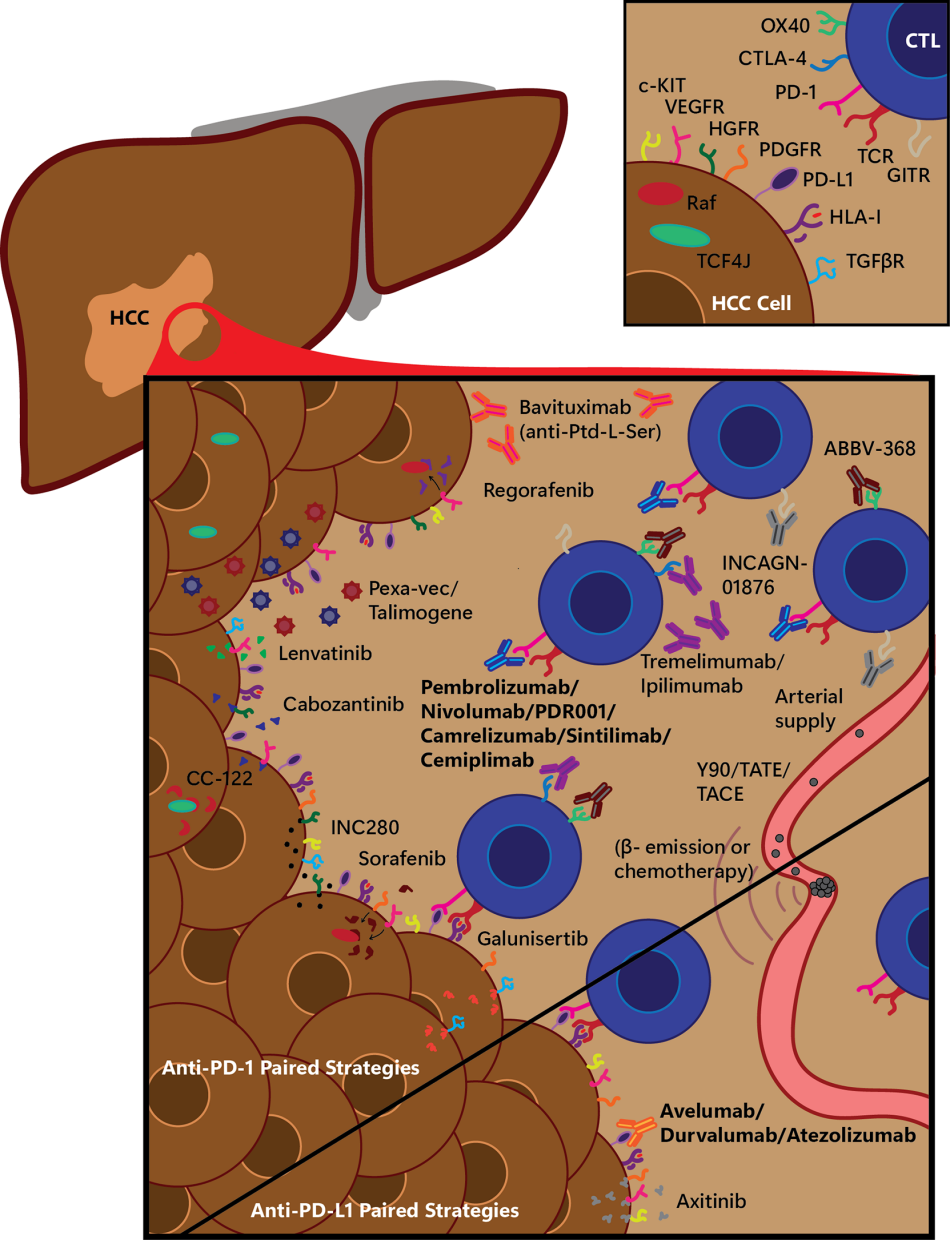
Selected experimental immunotherapy treatment combination strategies for anti-PD-1 and anti-PD-L1 therapy. Many small molecule drugs and other treatments are being tested in combination with standard immune checkpoint blockade (ICB) immunotherapeutics; a graphical overview of a selection of these are shown here, being tested with anti-PD-1 or anti-PD-L1 therapies. Main figure depicts various treatment strategies on a hepatocellular carcinoma (HCC) tumor, infiltrating cytotoxic T-lymphocytes (CTLs), and arterial-derived tumor-specific vasculature, divided into treatments being tested along with anti-PD-1 therapy (top, tested with either Pembrolizumab, Nivolumab, or PDR001) or with anti-PD-L1 therapy (bottom, tested with Avelumab, Durvalumab or Atezolizumab). β-, electron emission radiation; CTL, cytotoxic T-lymphocyte; CTLA-4, cytotoxic-T-lymphocyte-associated protein 4; HCC, hepatocellular carcinoma; HGFR, hepatocyte growth factor receptor; HLA-I, human leukocyte antigen class I; GITR, glucocorticoid-induced tumor necrosis factor receptor related; PD-1, programmed cell death protein 1; PD-L1, programmed cell death protein ligand 1; PDGFR, platelet-derived growth factor receptor; Pexa-Vec, pexastimogene devacirepvec; Ptd-L-Ser, phosphatidylserine; TACE, trans-arterial chemoembolization; TATE, trans-arterial tirapazamine embolization; TCF4J, transcription factor 4 J isoform; TCR, T-cell receptor; TGFβR, transforming growth factor β receptor; VEGFR, vascular endothelial growth factor receptor; Y90, yttrium-90 radioembolization.

Combination therapies with ICIs are a rising trend due to the modest activity of monotherapies in this disease. A combination of ICIs with multi-TKIs, conventional ablative therapies, or other immune modulating agents represent the next generation of HCC treatment modalities currently undergoing active investigation (**Figure 1**). Preclinical studies showed the potential synergistic efficacy of ICIs with TKIs and antiangiogenic drugs. Immunosuppressive properties of vascular endothelial growth factor (VEGF) have been demonstrated in preclinical models *via* inhibition of T-cell infiltration due to diminished permeability of tumor vessels (34, 35). Additionally, VEGF inhibits T-cell development and causes the upregulation of PD-1 and CTLA-4 expression on immune cells (36, 37). Although the clinical activity as single agent is modest, it has been shown that anti-angiogenic drugs could normalize the tumor vasculature and increase T cell infiltration by inducing the upregulation of the leukocyte adhesion molecules such as ICAM-1 and VCAM-1 on tumor endothelial cells (34). It was previously shown that VEGF inhibitor could synergize with ICIs in metastatic melanoma and there is an increase interest in combining anti-angiogenic treatments with ICIs to turn “cold’ tumors into “hot” tumors (38). Among other TKIs, cabozantinib’s potential effect of enhancing the response to ICIs (39) and lenvatinib`s immune-modulating activity when combined with PD-1 inhibitor were previously shown (40).

Recently, open-label, phase 3 IMbrave150 (NCT03434379) trial compared the efficacy of atezolizumab plus bevacizumab combination with sorafenib therapy in first line setting in 501 patients with advanced HCC. Atezolizumab plus bevacizumab showed significantly better OS at 12 months than sorafenib (67.2% vs. 54.6% respectively). Also, median PFS was 6.8 months in the combination arm while 4.3 months in sorafenib group (hazard ratio for disease progression or death, 0.59; 95% CI, 0.47 to 0.76; P<0.001) (41). Also, combination therapies targeting PD-1/PD-L1 and CTLA-4 are very effective in patients with malignant melanoma (42, 43), microsatellite instability high colorectal cancer, and are being investigated in many ongoing clinical trials in patients with HCC (44). The rationale for this strategy is based on the hypothesis that if CD8-positive T cells do not exist in cancer cells, blockade of the PD-1/PD-L1 pathway’s effectivity will be lower than expected. For this reason, blockade of the CTLA-4 can be an effective strategy to enhance the number of activated CD8-positive T cells that infiltrate the tumor cells. Based on preclinical data, many studies are currently evaluating the effectiveness of combination therapy with inhibitors against CTLA-4, PD-1, and PD-L1 in HCC (**Table 2**). The phase I/II CheckMate 040 trial (NCT01658878) investigated the effectiveness of the combination therapy of nivolumab and ipilimumab for the first time using various doses. On March 10, 2020, the FDA granted accelerated approval to the combination of nivolumab plus ipilimumab treatment for the patients with advanced HCC who have been previously treated with sorafenib. He et al. presented the results in the 2020 American Society of Oncology Gastrointestinal Cancers Symposium (ASCO-GI) Annual Meeting (45). The FDA approved the combination treatment based on promising ORR and duration of response (DOR). After a median follow-up of 30.7 months, 32% (16/49) of patients responded to combination treatment, with 8% (4/49) having a CR and 24% (12/49) having a PR (46). To date, this is the only dual immunotherapy approved by the FDA in the second-line setting treatment of HCC. Combination therapy with durvalumab and tremelimumab is being evaluated in a few ongoing clinical trials. An open-labeled, randomized phase I/II study (NCT02519348) testing this combination in patients with unresectable HCC appears safe and tolerable with a disease control rate of 57.5% (23/40 patients) and confirmed response rate of 15% (6/40 patients) (47). However, these promising results require further evaluation in a randomized setting and a larger patient population. Thus, an open-label, randomized, multicenter phase III clinical trial (NCT03298451) is being conducted to assess the efficacy and tolerability of the durvalumab plus tremelimumab combination therapy versus sorafenib as first-line treatment of patients with unresectable advanced HCC with results eagerly awaited.

**Table 2:** Ongoing clinical trials of combination therapy for immune checkpoint inhibitors in hepatocellular carcinoma (HCC).

Agents	Phase	Clinicaltrial.gov	Trial Name	Patient characteristics	Outcome
Nivolumab + Y90-Radioembolization	Phase 2	NCT03033446	–	Asian patients with advanced HCC	RR
Nivolumab + Cabozantinib	Phase 1	NCT03299946	CaboNivo	Following definitive resection in locally advanced HCC	AEs
Nivolumab + Regorafenib	Phase 1 & 2	NCT04170556	GOING	Patients with HCC who progressed after 1^st^ line therapy	AEs
Nivolumab + Regorafenib	Phase 2	NCT04310709	RENOBATE	Chemotherapy naïve patients with advanced HCC	RR
Nivolumab + Ipilimumab	Phase 1 & 2	NCT01658878	CheckMate-40	Systemic treatment naïve patients with advanced HCC	ORR, AEs
Nivolumab + Ipilimumab	Phase 3	NCT04039607	CheckMate-9DW	1^st^ line therapy in advanced HCC	OS
Nivolumab + Ipilimumab	Phase 2	NCT03222076	–	Neoadjuvant in patients with resectable HCC	AEs
Nivolumab + Abemaciclib	Phase 2	NCT03781960	–	Patients with inoperable HCC	ORR
Nivolumab + Ipilimumab	Phase 1 & 2	NCT03682276	PRIME-HCC	Preoperatively in patients with resectable HCC	Safety, Tolerability, Delay to surgery
Nivolumab + Ipilimumab	Phase 2	NCT03510871	–	Neoadjuvant therapy for the patients with non-metastatic HCC	Tumor shrinkage
Nivolumab + Ipilimumab + TACE	Phase 3	NCT04340193	CheckMate-74W	Patients with intermediate stage HCC	OS
Nivolumab + Relatlimab	Phase 1	NCT04658147	–	Perioperative treatment in potentially resectable HCC	Number of patients who proceed to surgery
Nivolumab + TACE	Phase 2	NCT03572582	IMMUTACE	Patients with intermediate stage HCC	ORR
Nivolumab + TACE	Phase 2 & 3	NCT04268888	TACE-3	Patients with intermediate stage HCC	OS
Nivolumab + Abemaciclib	Phase 2	NCT03781960	–	Patients with advanced HCC	ORR
Nivolumab + Lenvanitib	Phase 1	NCT03418922	–	Treatment naïve patients with HCC	DLTs, AEs
Nivolumab + Lenvanitib	Phase 2	NCT03841201	IMMUNIB	Patients with unresectable HCC	ORR, AEs
Nivolumab + Cabiralizumab	Phase 2	NCT04050462	–	Patients with unresectable HCC	ORR
Nivolumab + Sorafenib	Phase 2	NCT03439891	–	1^st^ line treatment in unresectable, locally advanced or metastatic HCC	MTD, ORR
Nivolumab + Tadalafil + Oral Vancomycin	Phase	NCT03785210	–	Patients with refractory advanced HCC	BOR
Nivolumab + BMS-986205	Phase 1 & 2	NCT03695250	–	1^st^ or 2^nd^ therapy in patients with advanced HCC	AEs, ORR
Nivolumab + Drug Eluting Bead TACE	Phase 1	NCT03143270	–	Patients with advanced HCC	AEs
TATE + nivolumab or pembrolizumab	Phase 2	NCT03259867	TATE-PD1	Patients with advanced HCC	RR
Nivolumab + ABBV-368	Phase 1	NCT03071757	–	Patients with locally advanced or metastatic HCC	Terminal half-life, MTD
Nivolumab + SF1126	Phase 1	NCT03059147	–	Patients with advanced HCC	DLTs
Nivolumab + APL-101	Phase 1 & 2	NCT03655613	–	2^nd^ line treatment in patients with locally advanced or	DLTs
Nivolumab + IRX-2	Phase 1	NCT03655002	–	2^nd^ line treatment or beyond in advanced HCC	RP2D, AEs
INCAGN01876 + Nivolumab or Ipilimumab	Phase 1 & 2	NCT03126110	–	Patients with advanced or metastatic HCC	Safety, tolerability, AEs, ORR
Pembrolizumab + Lenvatinib	Phase 3	NCT03713593	LEAP-002	1^st^ line therapy in advanced HCC	OS, PFS
Pembrolizumab + Lenvatinib + TACE	Phase 3	NCT04246177	LEAP-012	1^st^ line therapy in advanced HCC	OS, PFS
Pembrolizumab + Bavituximab	Phase 2	NCT03519997	–	Patients with advanced HCC	ORR
Pembrolizumab + Y90 Radioembolization	Phase 1	NCT03099564	HCRN GI15-225	Patients with poor prognosis HCC with preserved liver function	PFS
Pembrolizumab + TACE	Phase 1/2	NCT03397654	PETAL	Patients with advanced HCC	AEs
Pembrolizumab + SBRT	Phase 2	NCT03316872	–	Patients with advanced HCC	ORR
Pembrolizumab + Local ablation	Phase 2	NCT03753659	IMMULAB	Patients with early stage HCC	ORR
Pembrolizumab + Talimogene Laherparepvec	Phase 1/2	NCT02509507	MASTERKEY-318	Patients with advanced HCC	DLTs, ORR
Pembrolizumab + Regorafenib	Phase 2	NCT04696055	–	Patients with advanced HCC who have been previously treated with PD1/PDL1 inhibitors	ORR
Pembrolizumab + Regorafenib	Phase 1	NCT03347292	–	Patients with advanced HCC with no prior systemic therapy	TEAEs, DLTs
Pembrolizumab + Sorafenib Tosylate	Phase 1 & 2	NCT03211416	–	Patients with advanced HCC	ORR
Pembrolizumab + p53MVA vaccine	Phase 1	NCT02432963	–	Patients with solid tumors including HCC that have failed prior therapy	Tolerability
Atezolizumab + Cabozantinib	Phase 3	NCT03755791	COSMIC-312	1^st^ line therapy in advanced HCC	PFS, OS
Atezolizumab + Cabozantinib	Phase 1 & 2	NCT03170960	–	1^st^ line treatment in advanced HCC	ORR, MTD
Atezolizumab + Bevacizumab	Phase 3	NCT04102098	IMbrave050	Patients with HCC who has high risk of recurrence after resection or ablation	RFS
Durvalumab +/- Tremelimumab	Phase 3	NCT03298451	HIMALAYA	1^st^ line treatment in advanced HCC	OS
Durvalumab +/- Tremelimumab	Phase 2	NCT02519348	–	Immunotherapy naïve patients with advanced HCC	AEs, DLTs
Durvalumab + Tremelimumab	Phase 2	NCT03638141	–	Patients with intermediate stage HCC	ORR
Durvalumab + Cabozantinib	Phase 1 & 2	NCT02572687	CAMILLA	Advanced HCC progressed under at least 1 previous systemic therapy	ORR, MTD
Durvalumab + Tremelimumab +/- TACE	Phase 2	NCT02821754	–	Patients with HCC that progressed or intolerant to at least one line of therapy	PFS
Durvalumab + Tremelimumab + Radiation	Phase 2	NCT03482102	–	Patients with locally advanced, advanced or metastatic HCC	BOR
Durvalumab + TACE +/- Bevacizumab	Phase 3	NCT03778957	EMERALD-1	Patients with locoregional HCC	PFS
Durvalumab +/- Bevacizumab	Phase 3	NCT03847428	EMERALD-2	Patients with HCC who has high risk of recurrence after curative resection or ablation	RFS
Camrelizumab + Apatinib	Phase 2	NCT03463876	RESCUE	Patients with advanced HCC	ORR
Camrelizumab + Apatinib	Phase 3	NCT03764293	–	1^st^ line therapy in advanced HCC	OS, PFS
Camrelizumab + FOLFOX4	Phase 3	NCT03605706	–	1^st^ line therapy in advanced HCC	OS
Sintilimab + Bevacizumab biosimilar	Phase 2 & 3	NCT03794440	ORIENT-32	1^st^ line therapy in advanced HCC	OS. PFS
Anti-LAG-3 + REGN2810	Phase 1	NCT03005782	–	Patients with advanced solid tumors including HCC	AEs, DLTs
Anti-LAG-3 + Nivolumab	Phase 1 & 2	NCT01968109	–	Patients with advanced solid tumors including HCC	AEs, DCR
Anti-TIM-3 + LY3300054	Phase 1	NCT03099109	–	Patients with advanced/relapsed solid tumors including HCC	DLTs

TKIs and immune checkpoint inhibitors separately have proven efficacy in treating HCC. Thus, combined TKIs and immune checkpoint inhibitors have been investigated actively in recent clinical trials to obtain a better survival rate. Kudo et al. published results from a phase 1b study of avelumab plus axitinib as first-line treatment in patients with advanced HCC (NCT03289533). They reported that the avelumab plus axitinib combination showed antitumor activity (ORR: 31.8% [95% CI: 13.9-54.9%] per mRECIST for HCC) and was associated with a manageable toxicity profile (48). Other combination treatments of nivolumab plus sorafenib and nivolumab plus cabozantinib are under evaluation in phase I/II clinical studies (NCT03439891, NCT03299946). Combinations of pembrolizumab with either lenvatinib or regorafenib are similarly under evaluation in early-stage clinical trials. *In vitro* and *in vivo* preclinical studies of pembrolizumab plus lenvatinib combinations decreased TGF-ß and IL-10 by suppressing tumor-associated macrophages, Tregs, and related tumor microenvironment constituents (49). Preclinical work also demonstrated an effect on the downregulation of PD-1 and TIM3. Similar future studies are needed to identify the clear effects of the combined modalities of TKIs and immune checkpoint inhibitors. Ongoing combination therapies with ICIs and TKIs in clinical trials are summarized in **Table 2**.

Patients who received immunotherapy may develop immune-related adverse events (irAEs) due to their critical roles in self-tolerance. While a general perception is that ICI related to toxicities are milder than traditional cytotoxic and molecular targeted anticancer agents, some ICI-induced autoimmune conditions can be lethal, particularly if not immediately recognized (50). The most common immune-related adverse events in HCC patients who underwent anti-CTLA-4 treatment are skin rash, fatigue, and diarrhea (33). Treatment with anti-PD-1 antibodies causes fewer immune-related adverse events in patients with HCC than anti-CTLA-4 therapies. Reactivation of T-cells can cause cytotoxicity and an increase in hepatic damage. Withdrawal of ICI treatment and initiation of steroid therapy can control the vast majority of immune-related adverse events. There are well-established practical guidelines for evaluation and optimization of irAE management, including in populations of special interest (51, 52).

Second-generation immunotherapies such as TIM-3, LAG-3, and killer cell immunoglobulin-like receptor (KIR) are still in the relatively early stages of clinical development for advanced solid tumors (53). Combining these with anti-PD-1 antibody therapies represent novel ways to induce T-cell infiltration into the tumor microenvironment. Combinations of anti-PD1 therapies with LAG-3 (NCT03005782 and NCT01968109) and TIM-3 (NCT03099109) are currently in phase 1 clinical trials. Additional clinical trials will continue to assess the efficacy and safety of novel ICIs in treating advanced HCC.

## Immune Related Adverse Events of Combination Immunotherapies

With the development of cancer immunotherapy, the combination strategies are becoming prevalent. Moreover, immune-related adverse events (irAEs) occurred more frequently, showing a different pattern from single-agent therapy. In recent years, anti-PD-1 and anti-CTLA-4 antibodies combinations have been extensively studied, showing higher response rates than single-agent therapy. Previous studies showed that both the incidence and severity of irAEs increase with the combination of anti-CTLA-4 and anti-PD-1 therapy (54–56). A phase 3 CheckMate067 study showed that the incidence of grade 3 or 4 irAEs was 59% in the nivolumab plus ipilimumab combination group, 28% in the ipilimumab group 21% in the nivolumab group. The most common grade 3/4 irAEs were gastrointestinal reactions. Similarly, the Checkmat040 study showed an increased risk of irAEs in advanced HCC patients (57). Anti-PDL-1 plus anti-CTLA-4 combination immunotherapy also showed a higher irAEs profile. In a phase, I/II study of durvalumab plus tremelimumab combination and monotherapies tolerability was acceptable across arms. Grade 3 irAEs occurred 37.8% in combination arm while 20.8% in durvalumab arm (58). A combination of anti-PD-1 therapy with other immune checkpoint molecules is also under ongoing investigation. Previous studies showed that a combination of nivolumab plus LAG-3 was well tolerated. The most common irAEs were fatigue, diarrhea, and pruritis. Grade 3/4 irAEs occurred in 9% of patients (59).

To date, there are no immunotherapy combinations related to specific irAEs; however, the incidence of serious irAEs is higher than monotherapies. For grade 2 or above irAES, ASCO, and National Comprehensive Cancer Network (NCCN) practice guidelines suggest treatment interruption, close monitoring, supportive management (51, 60).

## Conclusion and Future Expectations

HCC is the second most frequent cause of cancer-related death worldwide, with limited therapeutic options. The immune system has an important and complex role in the pathogenesis of the HCC. Since the discovery of immune checkpoint proteins and development of their inhibitors, the treatment algorithms for HCC have dramatically changed and represent promising opportunities for patients. While ICIs have demonstrated promising results, efficacy remains limited for most patients and future improvements can only come through rigorous scientific investigations. We believe that ICIs will play an essential future role in the therapeutic interventions for patients with HCC, with the realization of improved clinical outcomes for all.

## Author Contributions

All authors contributed to the article and approved the submitted version. The corresponding author is responsible for submitting a competing financial interest statement on behalf of all authors of the paper.

## Conflict of Interest

The authors declare that the research was conducted in the absence of any commercial or financial relationships that could be construed as a potential conflict of interest.

## Publisher’s Note

All claims expressed in this article are solely those of the authors and do not necessarily represent those of their affiliated organizations, or those of the publisher, the editors and the reviewers. Any product that may be evaluated in this article, or claim that may be made by its manufacturer, is not guaranteed or endorsed by the publisher.
